# Evaluation of KRAS Concomitant Mutations in Advanced Lung Adenocarcinoma Patients

**DOI:** 10.3390/medicina57101039

**Published:** 2021-09-29

**Authors:** Veronica Aran, Mariano Zalis, Tatiane Montella, Carlos Augusto Moreira de Sousa, Bruno L. Ferrari, Carlos Gil Ferreira

**Affiliations:** 1 Instituto Estadual do Cérebro Paulo Niemeyer (IECPN), Rio de Janeiro 20231-092, Brazil; 2 Oncoclínicas, São Paulo 22251-060, Brazil; mariano.zalis@oncoclinicas.com (M.Z.); bruno.ferrari@oncoclinicas.com (B.L.F.); 3 Oncoclínicas, Rio de Janeiro 22251-060, Brazil; tatianemontella@gmail.com (T.M.); cgmferreira@gmail.com (C.G.F.); 4 Departamento de Tecnologias da Informação e Educação em Saúde (DTIES), da Faculdade de Ciências Médicas (FCM), na Universidade do Estado do Rio de Janeiro (UERJ), Rio de Janeiro 20550-170, Brazil; cam.sousa@bol.com.br

**Keywords:** KRAS, advanced lung adenocarcinoma, concomitant mutations, NGS

## Abstract

*Background and Objectives*: One of the most frequently mutated oncogenes in cancer belongs to the Ras family of proto-oncogenes, which encode distinct key signaling events. RAS gain-of-function mutations are present in ~30% of all human cancers, with KRAS being the most frequently mutated isoform showing alterations in different cancer types including lung cancer. This study aimed to investigate the incidence of KRAS mutations, and concomitant mutations, in advanced non-small cell lung adenocarcinoma patients. *Materials and Methods*: This was a retrospective study, where genomic DNA extracted from paraffin-embedded tumor tissues from 121 Brazilian advanced non-small cell lung adenocarcinoma patients were analyzed to evaluate via Next Generation Sequencing (NGS) the incidence of KRAS mutations and co-occurring mutations and correlate, when possible, to clinicopathological characteristics. Statistical analyses were performed to calculate the prevalence of mutations and to investigate the association between mutational status, mutation type, and sex. *Results*: The results showed a prevalence of male (N = 63; 54.8%) compared to female patients (N = 52, 45.2%), and mutant KRAS was present in 20.86% (24/115) of all samples. Interestingly, 33.3% of the mutant KRAS samples showed other mutations simultaneously. *Conclusions*: This study revealed the presence of rare KRAS concomitant mutations in advanced lung adenocarcinoma patients. Further investigation on the importance of these genomic alterations in patient prognosis and treatment response is warranted.

## 1. Introduction

Lung cancer kills approximately 1.8 million people worldwide every year, affecting more males than females [1]. The most frequently found histology subtype is non-small cell lung cancer (NSCLC), corresponding to 80% of tumors and subdivided into adenocarcinoma (35–40%), squamous cell carcinoma (25–30%), and large cell carcinoma (10–15%) [2]. The most common driving mutations observed in NSCLC correspond to the ones found in epidermal growth factor receptor (EGFR), KRAS, human epidermal growth factor receptor 2 (HER2), and EML4-ALK translocations. Mutations in KRAS have been shown in up to 30% of lung adenocarcinomas and in approximately 5% of the squamous-cell carcinoma subtype with a higher incidence occurring in current or former smokers than in never smokers [3]. The KRAS mutation frequencies in NSCLC vary according to different ethnic populations, ranging from 15–32% [4]. For example, a lower prevalence is observed in the Asian and Latin American populations (15%–20%) compared to the European populations, with the latter presenting higher prevalence (20%–30%) [5].

There are 3 RAS genes (KRAS, HRAS, and NRAS) frequently mutated in cancer with oncogenic mutations usually affecting codons 12, 13, and 61 [6]. KRAS is the most affected isoform and mutations are usually missense and primarily found in codons 12 and 13 of exon 2. These alterations produce aberrant activation of key effector cascades including the PtdIns 3-kinase (PI3K)-Akt pro-survival and the Raf-MEK-ERK proliferative pathways [7]. In non-small cell lung adenocarcinoma, mutations usually affect hotspots in codons 12, 13 (G12C > G12V > G12D > G13C > G13D), and codon 61 (Q61H > Q61L > Q61R) [6]. Besides lung, another cancer type that shows high frequency of KRAS mutations is colorectal cancer (CRC), with some studies indicating 40% rates [8]. Similarly to NSCLC, mutations usually affect hotspots in codons 12, 13 (G12D > G12V > G13C > G12C > G12A), and 61 (Q61H > Q61L > Q61R) [8,9].

Independently of the cancer type, mutant KRAS has been considered an undruggable target for more than three decades; nevertheless, new approaches for blocking KRAS continue to be developed [10,11]. The mutation G12C has been described as the most frequently altered in lung cancer, which is associated with poor prognosis and resistance to treatment. The prevalence of this mutation type varies among different countries, and the cysteine residue of the KRAS G12C has been exploited to design covalent inhibitors, which showed promising results, such as AMG 510 (now named sotorasib or LUMAKRAS™) and MRTX849 [12,13]. Both inhibitors act in a similar fashion by locking KRAS in an inactive GDP-bound state by binding the Cys12 residue in the KRAS switch II pocket. Sotorasib also includes aromatic rings that improve its potency relative to a previously reported compound (ARS-1620) [14], and in clinical trials, sotorasib demonstrated significant anti-tumor activity, thus becoming an option for advanced lung cancer patients for whom effective treatments are not available [13]. Recently, the FDA approval of sotorasib represented a major breakthrough in RAS personalized therapy [15]. In addition, different types of mutations may serve as predictors of non-responsiveness to targeted therapies (e.g., EGFR inhibitors in lung and colon cancer) [16,17].

It is well established that tumors may harbor mutations in different genes simultaneously. The presence of concomitant mutations may affect the response to targeted therapies, and this was confirmed in the case of EGFR tyrosine kinase inhibitors (EGFR-TKI) in NSCLC, where EGFR-mutant tumors also presented mutations in other genes (e.g., KRAS, BRAF, NRAS, MET), which were previously classified as mutually exclusive [18,19]. In addition, it was shown that KRAS mutations combined with mutations in EGFR or ALK rearrangements could negatively impact TKI response [20]. It has also been described that certain mutation frequencies may vary according to different characteristics such as sex [21]. We believe that the proper evaluation of the incidence of KRAS concomitant mutations in the Brazilian advanced non-small cell lung adenocarcinoma population is important for the global effort to understand the significance of KRAS mutations and their outcomes in response to distinct therapies, including the recent KRAS G12C-targeted drug sotorasib.

## 2. Materials and Methods

### 2.1. Patient Samples

This was a cross-sectional retrospective study. The data available belong to the private institute and clinic *Oncoclínicas*, Brazil. Data from 121 patients were analyzed to evaluate the incidence of KRAS G12C mutations in advanced lung adenocarcinoma patients and correlated, when possible, to clinicopathological characteristics such as sex. The inclusion criteria were patients diagnosed with advanced non-small cell lung adenocarcinoma independently of the clinical background (treated and naïve samples). The exclusion criteria included patients from whom it was not possible to obtain a report confirming the disease and patients from whom it was not possible to obtain molecular analysis due to insufficient amount of tissue sample or missing sample. This study was approved by the *Hospital Pró-cardíaco* ethics committee (*Hospital Pró-cardiaco*-Protocol: 4.109.474). Informed consent was waived because of the retrospective nature of the study and because the analysis used anonymous clinical data. All methods were carried out in accordance with relevant guidelines and regulations.

### 2.2. Sample Selection

FFPE tissues comprising advanced non-small cell lung adenocarcinoma were used and the amount of tumor in the analyzed sample ranged from 20–90%, as determined by a pathologist. In addition, nine reference samples were tested including four commercially available standards AcroMetrix™ Oncology Hotspot Control catalogue no. 969056. The limit of detection was calculated using data from the AcroMetrix™ Hotspot Frequency Ladder. The analyses were repeated using different DNA extractions from each patient in order to further confirm the results.

### 2.3. Nucleic Acid Extraction and Quantification

The DNA from FFPE tissues (five sections of 10 micra) was extracted using QIAmp DNA FFPE Tissue Kit (Qiagen, Hilden, Germany) following the manufacturer’s protocol. DNA concentrations were determined by fluorometric quantitation using Qubit 2.0 Fluorimeter with Qubit DNA dsDNA BR Assay Kit.

### 2.4. Next-Generation Sequencing

Library preparation was carried out using the Oncomine Assay™ (comprising the DNA Oncomine™ Focus Assay) (Thermo Fisher Scientific) following the manufacturer’s instructions, using a total of 10 ng input DNA per sample (minimum 0.83 ng/μL sample DNA concentration). A maximum of seven DNA samples were prepared per run on an Ion 318™ v2 chip (Thermo Fisher Scientific, catalog no. 4488150). The DNA panel can identify hotspot mutations in the following genes: AKT1, ALK, AR, BRAF, CDK4, CTNNB1, DDR2, EGFR, ERBB2, ERBB3, ERBB4, ESR1, FGFR2, FGFR3, GNA11, GNAQ, HRAS, IDH1, IDH2, JAK1, JAK2, JAK3, KIT, KRAS, MAP2K1, MAP2K2, MET, MTOR, NRAS, PDGFRA, PIK3CA, RAF1, RET, ROS1, and SMO; and K-RAS was the focus of this validation. Template preparation was performed on the Ion Chef System (Thermo Fisher Scientific, Waltham, MA, USA) using the Ion PGM Hi-Q Chef Kit and/or the Ion One Touch™ 2 System using the Ion PGM Template OT2 200 Kit. Sequencing was performed using the Ion PGM Hi-Q Sequencing Kit on the Ion Torrent Personal Genome Machine (Ion PGM).

### 2.5. Data Analysis

Analysis was carried out using Ion Torrent Suite™ Browser version 5.0 and Ion Reporter™ version 5.0. The Torrent Suite™ Browser was used to perform the initial quality control including chip loading density, median read length, and number of mapped reads. The Coverage Analysis plugin was applied to all data and used to assess amplicon coverage for regions of interest. Variants were identified by Ion Reporter filter chain 5% Oncomine™ Variants (5.0). A cutoff of 500× coverage was applied to all analyses.

### 2.6. Statistical Analysis

The statistical analysis was conducted using package stats from the R Statistical Software [22] to calculate the prevalence of patients who presented mutations, and the Chisq.test, which performs chi-squared contingency table tests and goodness-of-fit test, which was performed to evaluate the statistical association between sex and presence of mutations. The statistical significance level to determine if sex and mutations were correlated was 5%.

## 3. Results

### 3.1. Patient Characteristics

The study included 121 formalin-fixed, paraffin-embedded samples from advanced non-small cell lung adenocarcinoma patients. Six patients showed inconclusive results, whereas 115 had valid samples, which were analyzed for the incidence of KRAS mutations. Regarding sex, there was a prevalence of male (N = 63; 54.8%) compared to female (N = 52, 45.2%) patients in our study cohort.

### 3.2. Frequency of KRAS Alterations

**Figure 1:**
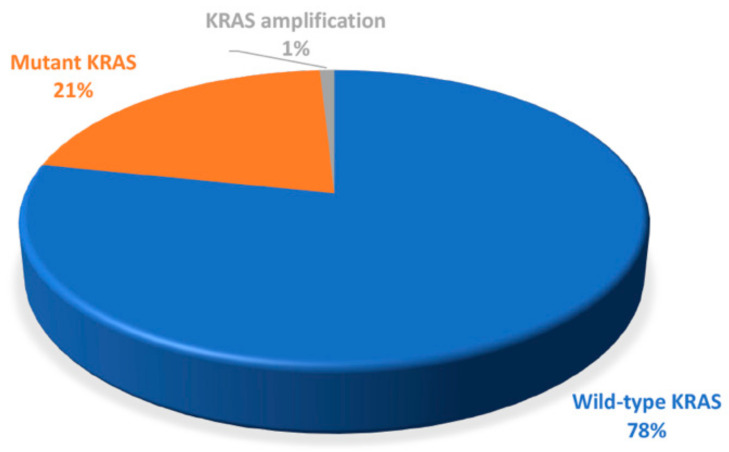
Percentage (approximate values) of mutant samples.

Patient samples were accessed for the incidence of KRAS alterations. We found that 21.7% of the valid samples (25/115) showed altered KRAS, where 24 patients presented KRAS mutations (20.87%) and one presented a KRAS amplification (0.87%). The total approximate values are shown in Figure 1. Mutations were present in exons 2, 3, and 4, and predominantly found in codon 12 (18/115) followed by codon 13 (3/115), codon 61 (3/115), and one mutation in codon 146 (1/115).

### 3.3. Types of K-RAS Mutations

**Figure 2:**
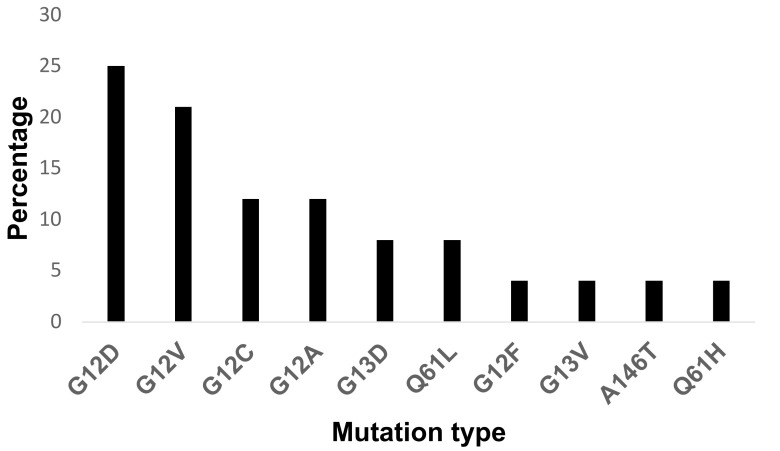
Prevalence of different KRAS mutations in advanced adenocarcinoma patients.

Of the mutant samples, the most frequent mutation types were G12D (6/24), followed by G12V (5/24), G12C/G12A (3/24), G13D/Q61L (2/24), and G12F/G13V/A146T/Q61H (1/24). Figure 2 summarizes the percentages of each mutation in relation to the total number of mutant samples.

### 3.4. KRAS Concomitant Mutations

KRAS concomitant mutations were detected in eight of 24 mutant samples (33.3%), where three (3/8) of them occurred concomitantly with the mutation G12D and two occurred concomitantly with the G12V mutation (2/8). The remaining mutations occurred only once with KRAS p.A146T, G12C, and G13D. One patient harbored two co-occurring KRAS mutations (G12D and Q61L). The tumors that showed KRAS concomitant alterations are shown in Table 1, and the results indicated no specific prevalence pattern.

### 3.5. Analysis of Patient Sex Versus KRAS Status

Previous studies have shown that lung cancer incidences and mutations may be linked to distinct clinicopathological characteristics such as sex. Thus, when sex was analyzed, the majority were male (63/115), and there was no association between sex and the KRAS mutational status (*p* value = 0.92), as shown in Table 2. In addition, the analysis on the prevalence of KRAS concomitant mutations according to sex revealed no statistical association (*p* value = 0.93).

## 4. Discussion

The driver genes’ heterogeneity present in tumors may lead to clonal and subclonal populations of cells that arise in different times in tumor evolution [23]. Simultaneous genomic changes may impact biological behaviors, such as treatment response; thus, the investigation of co-occurring genomic alterations could help to stratify KRAS-mutant lung cancer patients into distinct subgroups with distinctive therapeutic responses [24]. While driver mutations (e.g., mutations in KRAS, EGFR, BRAF) are known to initiate tumor development in lung cancer, secondary mutations may promote subclonal evolution such as mutations in TP53 and PIK3CA, which were previously described in KRAS-, BRAF-, and EGFR-mutant lung tumors [25,26]. Scheffeler and colleagues conducted a study on 4507 NSCLC patients using a NGS panel containing 14 genes, and 53.5% of the mutant KRAS patients had at least one additional mutation, a percentage higher than ours, since KRAS concomitant mutations were observed in eight of 24 mutant samples (33.3%) [27]. Similarly to the present study, their results also identified KRAS concomitant mutations affecting the genes KIT, CTNNB1, PIK3CA, and MET, also indicating that some of these events are under clinical evaluation [27]. Most of the concomitant mutations observed in this study were double mutations (7/8) and one patient harbored five simultaneous alterations, which could be considered a rare event since double or triple mutations are more frequently found [19].

The frequency of the distinct types of KRAS mutations in lung adenocarcinoma vary, with G12C, G12V, and G12D being the most frequently observed among different studies worldwide [28]. In our Brazilian cohort, we also identified these mutations as the most frequent in addition to G12A. A recent study showed that KRAS G12C mutations occur between 3–14% of different cancer types including NSCLC, colorectal cancer, appendiceal, and small bowel cancer [21], suggesting also that, in NSCLC, female patients harbored significantly more KRAS G12C mutations than male patients [21]. In our cohort, 2.6% of the total samples had KRAS G12C mutations, representing 12.5% of all KRAS-mutant samples. There was no significant association between the findings and sex, which was also previously observed [29].

Patients harboring KRAS G12C mutations can benefit from the inhibitor sotorasib [12,30]; therefore, it is important to investigate the possibilities of secondary mutations being able to potentially interfere with drug therapy responses. For example, a Chinese study found that some of the most predominant KRAS G12C concomitant mutations affected the genes EGFR, ROS1, and MET [28]. In our study, only one of the eight co-occurring mutations occurred alongside KRAS G12C, with MET being the affected gene (KRAS p.G12C; MET p.T1010I). Probably in this scenario, a combinational therapy developed to target both MET and KRAS G12C could be an option to avoid the possibility of therapy resistance. Indeed, the MET pathway was shown to be upregulated in KRAS-mutant lung cancer after MAPK inhibition and after treatment with KRAS inhibitors [31,32], an effect that could be enhanced by mutant MET.

**Figure 3:**
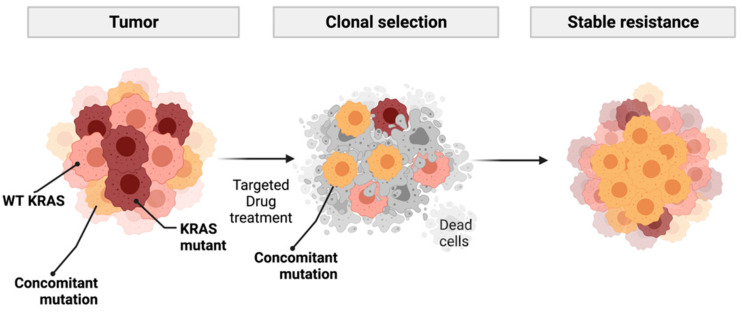
Schematic representation of possible subclonal evolution and drug resistance in tumors harboring KRAS concomitant driver mutations. Created with BioRender.com.

Our analysis also identified rare co-occurring mutations. For example, one patient harbored mutations in the genes IDH1 and KRAS (KRAS G12V; IDH1 R132L), which could represent an evidence of subclonal evolution, as previously described [26]. In addition, IDH1/2 mutations in lung adenocarcinoma are considered uncommon events [26,33]. Additionally, we also identified a patient with a KRAS G12D mutation and a ROS1 gene fusion [34], a finding that was previously described as a rare event [34,35,36]. The real relevance of these mutations awaits further investigations since they might interfere with ROS1 signaling, which could impact ROS-targeted therapies such as crizotinib. Although the role of KRAS co-occurring mutations remains to be clarified, it has been described that these alterations may occur clonally or subclonally in lung cancer, a fact that may affect treatment responses [23]. Figure 3 shows a schematic representation of the possible consequence of the subclonal evolution of cells containing concomitant mutations that may confer resistance to target therapies. These therapies are usually designed to target one mutant gene, and subclones may contain other important mutations able to generate proliferative signals, which are not blocked by the target therapy leading to drug resistance.

As the presence of concomitant mutations were shown to affect the activity of first-line EGFR TKIs in a subgroup of EGFR mutant NSCLC tumors [18], the same could occur in patients harboring the KRAS concomitant mutations found in the present study. Therefore, more studies on larger cohorts are necessary to also evaluate treatment responses in patients harboring distinct KRAS concomitant mutations including the ones described here. Current treatment protocols may change depending on the results observed.

## 5. Conclusions

The presence of concomitant mutations in the cohort analyzed suggests that each individual may present distinct therapeutic vulnerabilities depending on their tumor’s mutational spectrum. KRAS testing alongside the identification of other affected genes in the same patient will make the treatments even more personalized by contributing more accurately to the clinical decision process. Furthermore, the genomic stratification of each lung cancer type into distinct molecular subtypes will further improve this scenario. Even though specific targeted therapies are being developed to act upon the different lung cancer mutational spectra, how the different treatments may impact different populations across the globe, and the tumors harboring distinct concomitant mutations, remains to be determined.

## Figures and Tables

**Table 1:** List of KRAS concomitant mutations identified.

Patient Sex	Mutation
Male	KRAS p.G12D; KRASp.Q61L
Male	KRAS p.G12D; ROS fusion (SLC34A2-ROS1)
Female	KRAS p.G12V; IDH1 p.R132L
Female	EGFR p.L747_A750delinsP, KRAS p.A146T; PIK3CA p.E81K; MTOR p.E1799K; SMO p.R209C
Male	KRAS p.G12D; PIK3CA p.E545K
Female	KRAS p.G13D; KIT p.V825I
Male	KRAS p.G12C; MET p.T1010I
Male	KRAS p.G12V; CTNNB1 p.S33Y

**Table 2:** Patient sex versus KRAS status.

Altered KRAS						Total		*p* Value *
		**No**	**%**	**Yes**	**%**		**%**	
Sex	Female	40	34.8	12	10.4	52	45.2	0.92
	Male	50	43.5	13	11.3	63	54.8	
Total		90	78.3	25	21.7	115	100	
**Concomitant KARAS Mutations**						**Total**		
		**No**	**%**	**Yes**	**%**		**%**	
Sex	Female	49	42.6	3	2.6	52	45.2	0.93
	Male	58	50.4	5	4.3	63	54.8	
Total		107	93.0	8	7.0	115	100.0	

* Pearson’s Chi-squared test with Yates’ continuity correction.

## Data Availability

The data sets used and/or analyzed during the current study are available from the corresponding author on reasonable request.
